# Integrative rehabilitation in the treatment of lumbosacral muscle strain in elite trampoline athletes: a pilot study

**DOI:** 10.3389/fspor.2024.1383228

**Published:** 2024-07-09

**Authors:** Jia-Yin Ma, Jia-Jia Wu, Jing Zhang, Qing Zhao, Feng-Tao Shen, Ling Feng, Guo-Hui Zhang, Yi Zhu, Jian-Guang Xu

**Affiliations:** ^1^ Center of Rehabilitation Medicine, Yueyang Hospital of Integrated Traditional Chinese and Western Medicine, Shanghai University of Traditional Chinese Medicine, Shanghai, China; ^2^ Engineering Research Center of Traditional Chinese Medicine Intelligent Rehabilitation, Ministry of Education, Shanghai, China; ^3^ Department of Traditional Chinese Medicine, General Hospital of Eastern Theater Command, Nanjing, China; ^4^ NHC Key Laboratory of Hand Reconstruction, Fudan University, Shanghai, China; ^5^ Shanghai Key Laboratory of Peripheral Nerve and Microsurgery, Shanghai, China; ^6^ Institute of Hand Surgery, Fudan University, Shanghai, China; ^7^ Department of Hand Surgery, Huashan Hospital, Fudan University, Shanghai, China

**Keywords:** lumbosacral muscle strain, low back pain, rapid arm-rise test, internal postural interference, physical therapy, traditional Chinese medicine, elite athletes

## Abstract

**Background:**

Lumbosacral muscle strain (LMS) is common in Chinese elite trampoline athletes. Advanced lumbar muscle activation is necessary for postural control before upper extremity voluntary movements, called anticipatory postural adjustment to reduce internal postural interference (IPI). The potential of delayed lumbar muscle activation has been reported in patients with non-specific LBP (NLBP) in response to IPI. However, it remains unknown whether this effect exists in elite trampoline athletes. There is also limited literature reporting the rehabilitation of LMS in this population. This study first aimed to explore whether elite trampoline athletes with LMS experience delayed activation of lumbar muscles under IPI. The secondary aim was to preliminarily evaluate an integrative rehabilitation program's effectiveness.

**Materials and methods:**

Ten elite trampoline athletes with LMS were recruited and received 10 sessions of integrative rehabilitation, including extracorporeal shock wave therapy, acupuncture, Tui-na, and spine function exercises. At baseline and after all sessions, the relative activation time of the lumbar muscles under IPI in a modified rapid arm-rise test was used as a primary outcome measure. The secondary measures included a visual analog scale (VAS) and a questionnaire to assess low back pain (LBP) and athletic training performance.

**Results:**

The relative activation time of the lumbar muscles under IPI was delayed at baseline, but significantly decreased after the intervention (*P* < 0.05). The VAS was significantly decreased after the intervention (*P* < 0.05). There was no significant correlation between the difference in VAS and in activation time of the lumbar muscles before and after the intervention (*P* > 0.05).

**Conclusions:**

Elite trampoline athletes with LMS had delayed activation in their lumbar muscles under IPI. Integrative rehabilitation was effective in LBP relief and neuromuscular control of the lumbar muscles, and impacted positively on training performance. Future studies with a larger sample size, a control group, and long-term follow-ups are needed to further examine the efficacy of integrative rehabilitation in elite trampoline athletes with LMS. Additionally, the application of this approach in athletes with LMS or LBP in other sports, particularly those involving IPI, should be explored.

## 1 Introduction

Lumbosacral muscle strain (LMS) is very prevalent, often resulting from isolated traumatic incidents or repetitive overuse of the lumbar muscles (1). Trampolining is a highly competitive and advanced sport, which requires athletes to perform complex acrobatic skills involving multiple somersaults and twists in the air, with the torso in various positions. These skills require significant involvement of the core area of the body (2). The rate of lumbar injuries of elite trampoline athletes in China is up to 83.3%, of which LMS are responsible for 51.7% (2). LMS significantly affects these athletes' physical health and performance (3).

**Figure 1:**
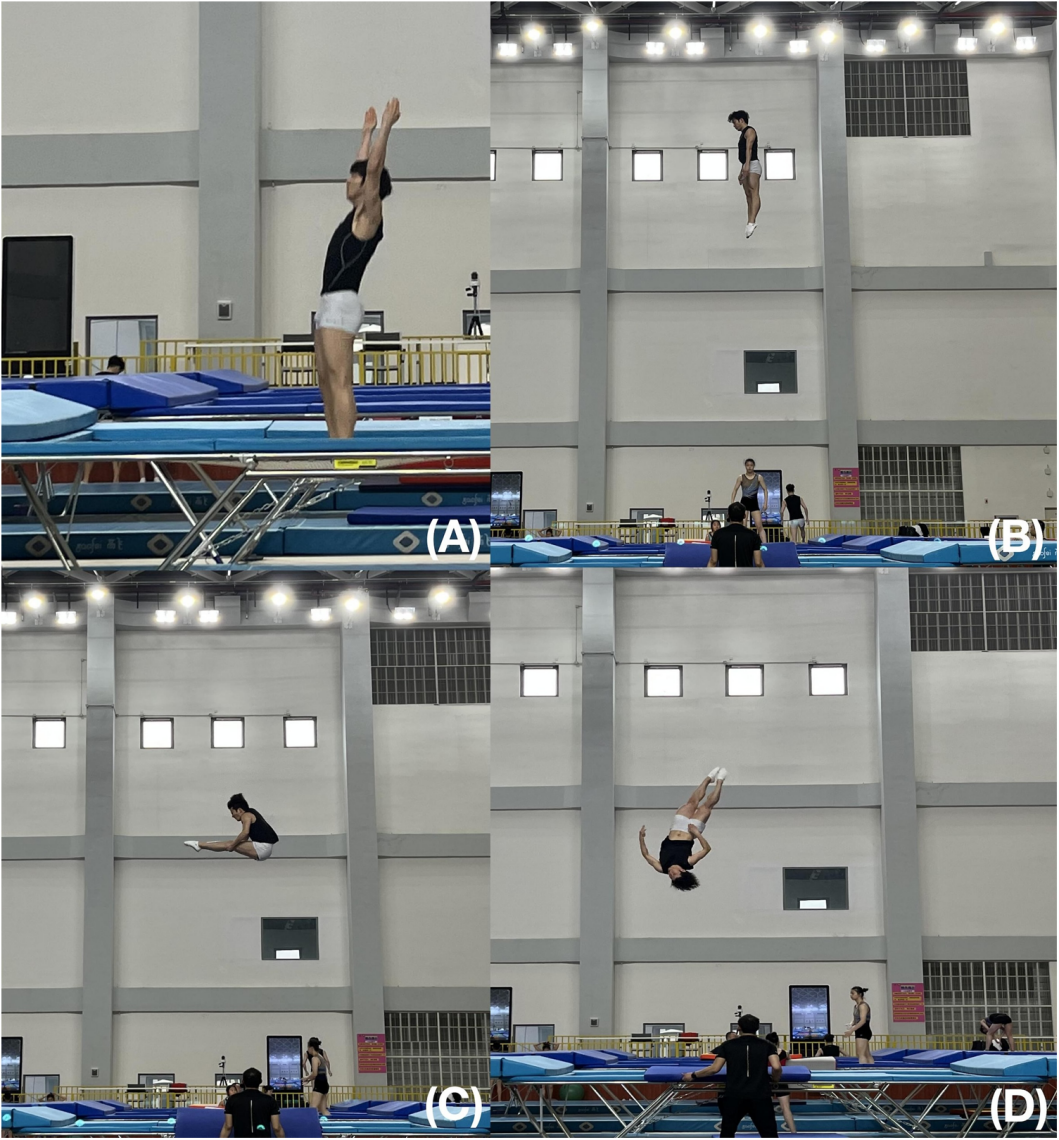
Trampoline acrobatic skills. (**A**) Rapidly rising bilateral arms as preparatory movement; (**B**) jumping up from the net; (**C,D**), examples of acrobatic skills.

In addition to lower back pain (LBP), LMS leads to decreased neuromuscular control of the core (4), which is essential for competitive trampoline sports (2). Trampoline acrobatic skills involve substantial feedforward control of the athlete's core when lifting both arms to jump from the net to a certain height and then perform acrobatic skills (3) (Figure 1). Under these conditions, voluntary extremity movements such as internal postural interference (IPI) require anticipatory postural adjustment of the core including the lumbar muscles (4). The potential of delayed lumbar muscle activation has been reported in patients with non-specific LBP (NLBP) in response to IPI (5). Therefore, we hypothesized that elite trampoline athletes with LMS also experience delayed lumbar muscle activation under IPI, which could result from their repetitive and intensive athletic training, and also reversely result in affecting their physical health and athletic training performance.

Rehabilitation of LMS in elite trampoline athletes has received little attention, despite the significant need. We previously offered long-term rehabilitation medicine service to the Shanghai trampoline sport team athletes, applying an integrative rehabilitation program that integrates multiple intervention methods of Western medicine and traditional Chinese medicine (TCM) for LMS rehabilitation. As integrative rehabilitation is more effective than single intervention methods for LMS (6), we hypothesized that an integrative rehabilitation program would have positive effects on the LMS of elite trampoline athletes.

LMS affects not only the physical health of elite trampoline athletes, but also their athletic training and competitive performance (2, 3). Previous studies have focused on neuromuscular control of common LMS patients (4, 5, 7–9), but there have been few studies conducted on elite trampoline athletes with LMS. According to the characteristics of trampoline sports and specific sports injuries in the lumbar muscles, targeted rehabilitation evaluation and treatment of elite trampoline athletes is necessary and of great importance (2, 3, 10).

We, therefore, aimed to explore whether elite trampoline athletes with LMS manifest delayed activation of lumbar muscles under IPI. We applied an integrative rehabilitation program for elite trampoline athletes with LMS, including extracorporeal shock wave therapy (ESWT), acupuncture, Tui-na, and spinal function training. The secondary aim of this study was to explore the therapeutic effects of this rehabilitation in elite trampoline athletes with LMS regarding relieving pain, improving neuromuscular control of the lumbar muscles, and enhancing athletic training performance.

## 2 Materials and methods

### 2.1 Study design

This was a single-arm, pre- and post-measurement design pilot study, to explore whether elite trampoline athletes with LMS experience delayed activation of lumbar muscles under IPI and to evaluate the effectiveness of an integrative rehabilitation program. The study was a collaborative effort between Yueyang Hospital of Integrated Traditional Chinese and Western Medicine affiliated with Shanghai University of TCM and Shanghai Chong-ming Sports Training Base. The study protocol was reviewed and approved by the Ethics Committee of Yueyang Hospital of Integrated Traditional Chinese and Western Medicine affiliated with Shanghai University of TCM (approval number: 2020-015), and was registered with the Chinese Clinical Trial Registration Center (No. ChiSTR2000034691).

The integrative rehabilitation program included ESWT, acupuncture, Tui-na, and spine function exercises. The intervention of ESWT and spine function exercises was performed by a certified physical therapist, whereas the intervention of Tui-na and acupuncture was performed by a certified physician of TCM. The intervention followed the participants' daily training routine. The outcome variables were measured by an experienced evaluator. The interventions were performed in a therapy room in the Sports Training Base. The physical therapist and the TCM physician performed their treatment at different times, while the evaluator performed baseline and post-intervention assessments independently of both.

### 2.2 Participants

According to a prior study, the relative activation time of the left multifidus to the anterior deltoid was considered a variable in the sample size analysis (5). Considering a type I error of 5% and a power of 80%, with a statistical significance *α* = 0.05 and an effect size of 0.10, a sample size of at least 10 participants was determined using G*Power 3.1.9.7®.

The diagnostic criteria of LMS were based on The Criteria for Diagnosis and Therapeutic Effect of Diseases and Syndromes in TCM by the State Administration of Traditional Chinese Medicine (11), and included the following: (1) long-term, recurrent occurrence of LBP; (2) lumbosacral pain and discomfort on one or both sides, which may range from mild to severe, and worsens with exertion and relieves with rest; (3) tenderness in the sacrospinalis muscles of one or both sides; and (4) no movement dysfunction of the low back and lower limbs. LBP referred to chronic non-specific LBP, which was defined as pain, muscle tension, and stiffness between the 12th rib and inferior gluteal fold lasting for at least 12 weeks, in the absence of radiculopathy, specific spinal diseases, or nerve root pain (12, 13).

The participant inclusion criteria were as follows: (1) age ≤ 30; (2) trampoline athlete of the Shanghai trampoline sports team; (3) meeting the diagnostic criteria for LMS (11); (4) LBP lasting for at least 12 weeks (12, 13); (5) not having received drugs (e.g., analgesics, non-steroidal anti-inflammatory drugs) or related interventions within 2 weeks; and (6) being informed and agreeing to complete the intervention as required. We excluded those with pain and discomfort in the lower back caused by musculoskeletal diseases (e.g., lumbar disc herniation, spinal fracture, severe arthritis, bony spinal stenosis, ankylosing spondylitis), internal medicine, or gynecological diseases; muscle pain extending down the leg toward the foot (13); (3) with fever, skin ulceration, infection, and other conditions that could affect evaluation or intervention; and (4) those undergoing any other treatments other than this protocol during the study.

**Figure 2:**
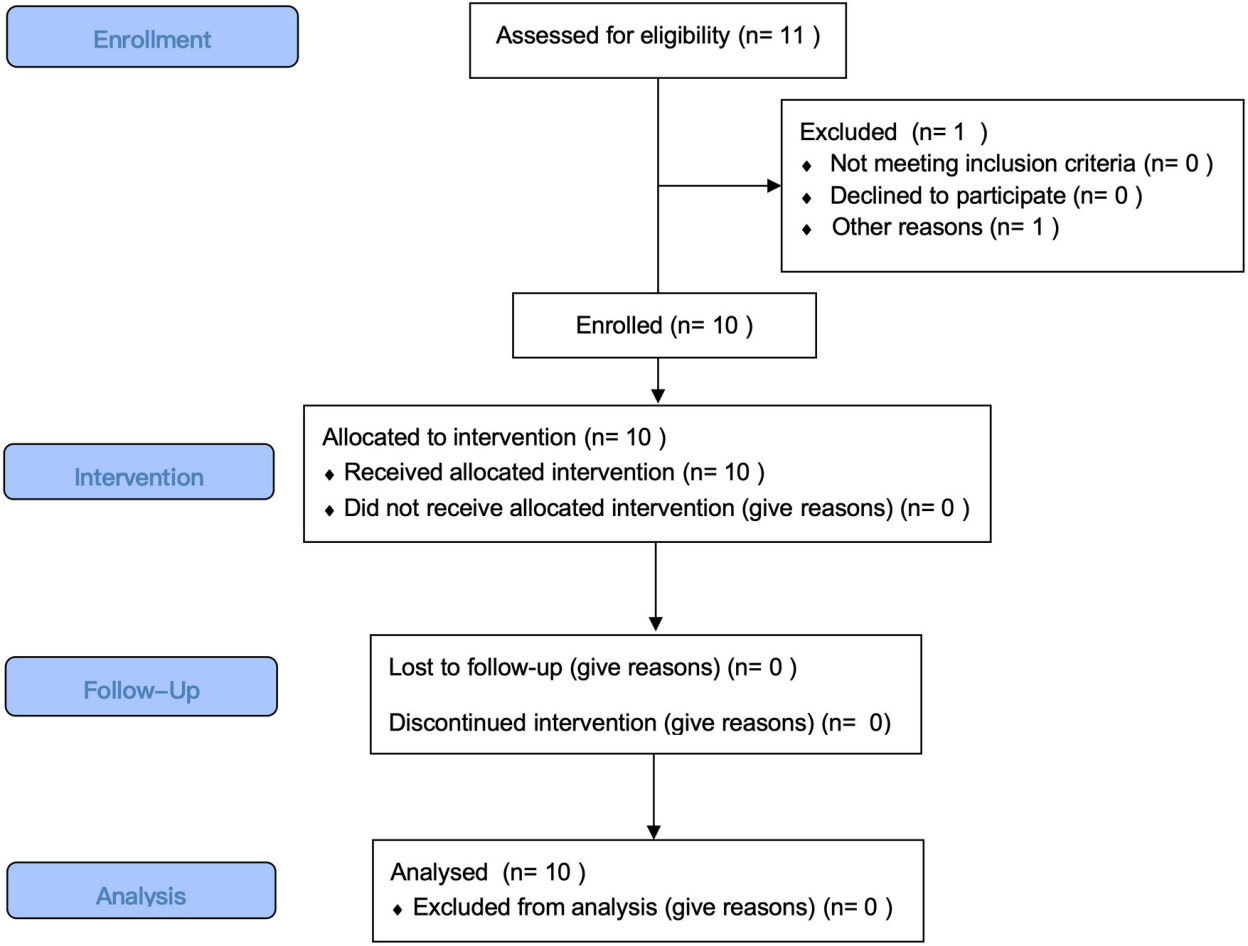
Consort flow diagram.

Recruitment was conducted within the Shanghai trampoline sports team, which initially consisted of 11 athletes meeting the inclusion criteria. However, one athlete was unable to participate in the full intervention and assessment process due to commitments outside of Shanghai, and therefore was not included in the study. Consequently, a total of 10 athletes (seven males, three females) were enrolled. These athletes voluntarily agreed to participate in the study and signed informed consent forms. The Consort flow diagram is illustrated in Figure 2 and the demographic information is described in Table 1.

**Table 1:** Demographics of the participants.

	Baseline (*n* = 10)
Gender (number)	
Male	7
Female	3
Age (year)	23.00 (18.50, 24.25)
Training period (years)	18.00 (13.75, 19.25)
History of LBP (years)	2.00 (2.00, 3.25)
Height (cm)	170.00 (163.00, 170.75)
Weight (kg)	61.50 (49.50, 70.00)

LBP, low back pain.

### 2.3 Procedures

#### 2.3.1 Integrative rehabilitation

During the integrative rehabilitation, the participants received a combination of ESWT for 5 min, acupuncture for 20 min, Tui-na for 20 min, and spinal function training for at least 20 min. The intervention was conducted every other day with a total of 10 sessions, lasting for 16 days. Each session required at least 65 min.

##### 2.3.1.1 ESWT

ESWT is a non-invasive medical treatment that uses high-energy shock waves to treat musculoskeletal disorders and chronic pain. The Shockmaster 500 device (Storz Medical, Switzerland) was used to deliver the ESWT intervention.

**Figure 3:**
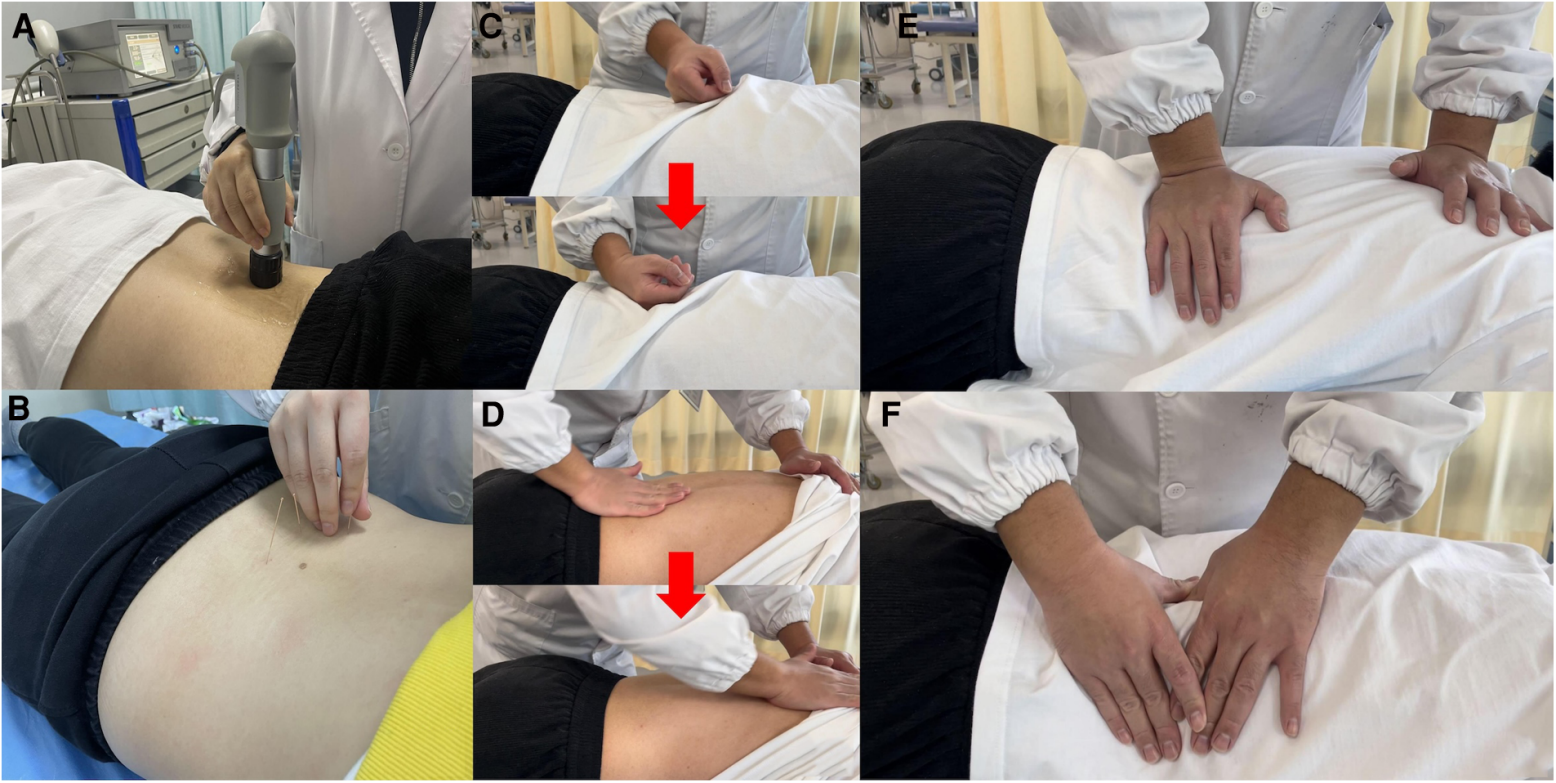
Integrative rehabilitation therapy. (**A**) Extracorporeal shock wave therapy (ESWT); (**B**) Acupuncture; (**C**) rolling technique of Tui-na (the red arrow showed the process); (**D**) rubbing technique of Tui-na (the red arrow showed the process); (**E**) kneading technique of Tui-na; (**F**) plucking technique of Tui-na.

The therapy was administered by a certified PT on a therapy bed using a contact method, with the labile movement of the hand-held applicator (head) in the spinal region of the trigger points at the level of the lumbar and sacral spine reported by the participants. A standard ultrasound gel was used on the applicator head in contact with the skin, to reduce tissue resistance and maintain proper coupling and energy propagation. The ESWT intervention consisted of delivering 2,000 total shocks for approximately 5 min in each session, with a frequency of 8 Hz and pressure of 2.0–2.5 Bar (Figure 3A).

##### 2.3.1.2 Acupuncture

Acupuncture is a TCM therapy that involves the insertion of thin needles into specific points on the body known as acupoints. The certified TCM physician was responsible for delivering acupuncture sessions individually to the participants in a prone position on a therapy bed. The skin around the acupoints was disinfected with 75% alcohol before the needles were inserted. The needles used were disposable sterile stainless-steel needles (Huatuo acupuncture needle, Suzhou Medical Appliance, Jiangsu, China) with a length of 50 mm and a diameter of 0.30 mm. Acupuncture point selection and specific operation methods were based on the Evidence-based Acupuncture Clinical Practice Guidelines for Low Back Pain of the China Association of Acupuncture-Moxibustion (14). “Jiaji” acupoints in areas of discomfort reported at the level of the lumbar and sacral spine by the participants were used as the main acupoints, and bilateral “Shen shu” (BL23), “Qi hai shu” (BL24), “Da chang shu” (BL25), “Guan yuan shu” (BL26), “Zhi bian” (BL54), “Huan tiao” (GB30), “Wei zhong” (BL40), and “Kun lun” (BL60) were used as secondary acupoints (15, 16). With the patient in the prone position, the needles were inserted 5–7 mm into each acupoint. The needles were twirled, lifted, and thrusted for elicitation of “de qi” (numbness and soreness) sensation. The needles were retained for 20 min before being withdrawn (14) (Figure 3B).

##### 2.3.1.3 Tui-na

Tui-na intervention involves a series of massage techniques applied to specific areas of the body, including the lower back and limbs. The certified TCM physician delivered Tui-na sessions individually to the participants. Relaxation techniques were used, including rolling (Figure 3C), rubbing (Figure 3D), kneading (Figure 3E), and plucking (Figure 3F), to relax the lumbar muscles, including the erector spinae, gluteus medius, gluteus maximus, piriformis, and tensor fascia lata. The meridians in the lower limbs, including the gallbladder meridian of foot-shaoyang and the bladder meridian of foot-taiyang were also included. Each Tui-na session lasted for 20 min.

##### 2.3.1.4 Spine function exercises

Spine function exercises were instructed and supervised by the certified PT. The training included thoracic spine and cat-arching exercises for spine stretching, and bird-dog and dead bug exercises for lumbar muscle strengthening (Supplementary Material S1). These exercises targeted improved spinal function and lasted for 5 min each.

### 2.4 Outcome measures

The study used three outcome measures to evaluate the effect of the intervention. The relative activation time of the lumbar muscles under IPI in a modified rapid arm-rise test (RART) was used as a primary outcome measure, while the secondary outcome measures included a visual analog scale (VAS) and a questionnaire on athletic training performance.

#### 2.4.1 Modified rapid arm-rise test (RART)

**Figure 4:**
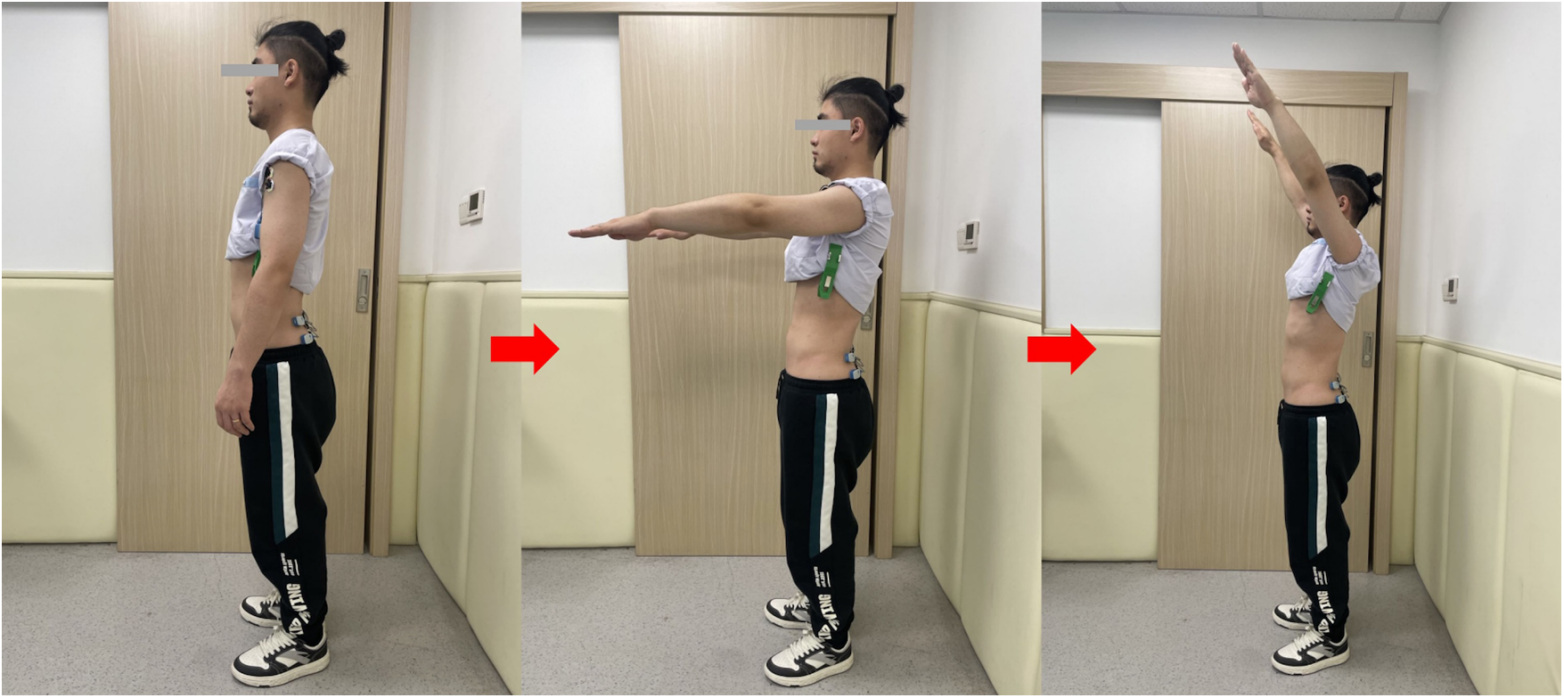
Process of the modified rapid arm-rise test. The red arrow shows the order of the process.

RART is a classic test assessing feedforward control of lumbar muscles under IPI, which reflects anticipatory postural adjustments of core muscles including lumbar paraspinal muscles in the condition of trunk perturbation. Trunk perturbation occurs internally and spontaneously when the arm rises rapidly (15). RART can be used to evaluate if lumbar muscles delay activating responses to IPI (15). Trampoline sports involve feedforward control of the athletes' lumbar muscles under IPI; therefore, we innovatively modified this test to simulate the characteristics trampolining, to evaluate this specific population. We changed the IPI from right rapid arm lifting to bilateral rapid arm lifting (Figure 4). Surface electromyography (sEMG) was used to record the activation time of specific muscles during the test. The test was evaluated by an experienced evaluator (17).

##### 2.4.1.1 Evaluation equipment

To record sEMG, self-adhesive bipolar Ag-AgCl surface electrodes and a 16-channel telemetry electromyography DTS system (Noraxon Inc.), with a sampling frequency of 1,500 Hz, CMRR of <−100 DB, and noise of <1 µV, were used. The signals were converted by a 24-bit analog-to-digital converter and stored in a computer for processing.

##### 2.4.1.2 Evaluation procedure

**Figure 5:**
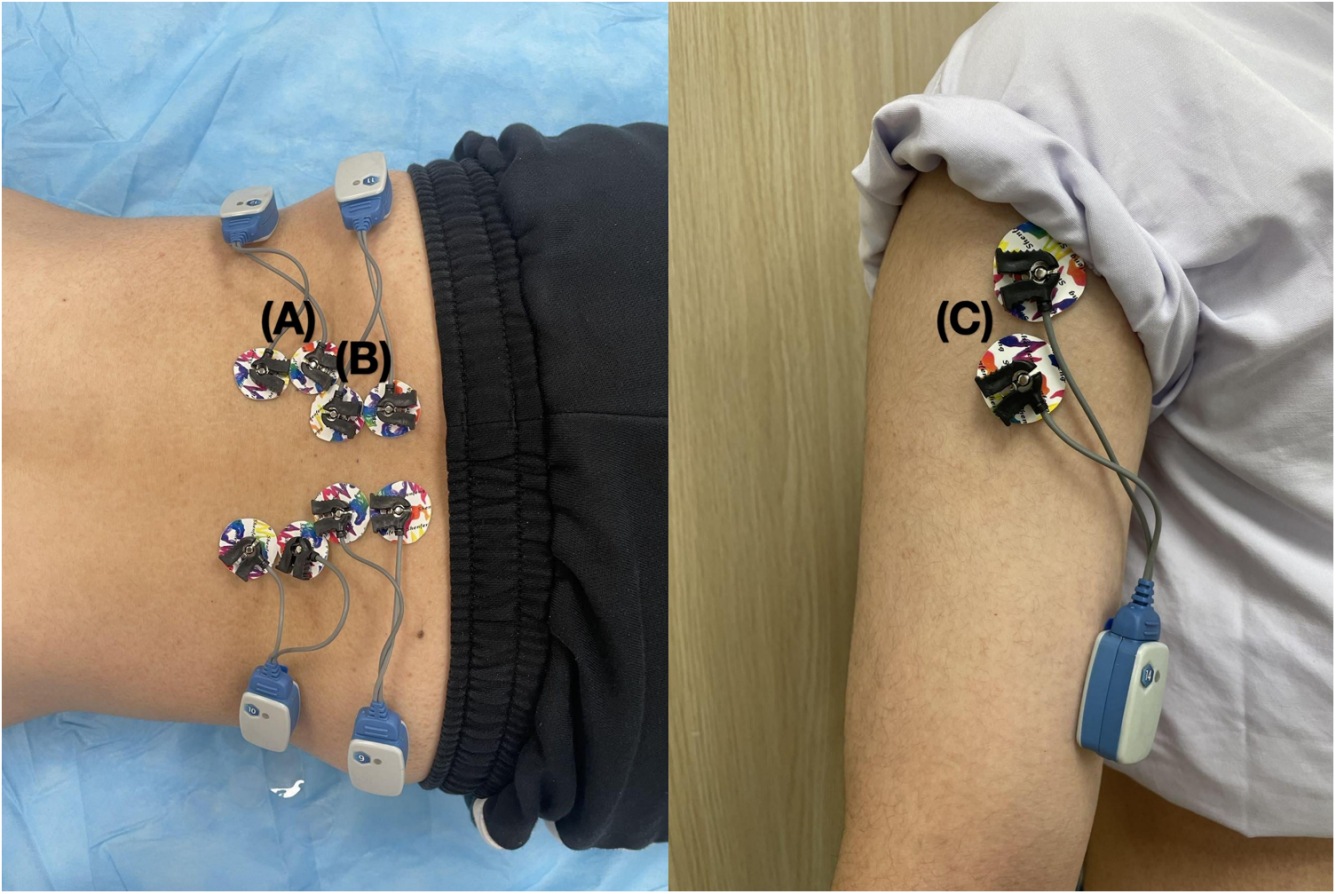
The electrode placement in modified RART. Left: The electrode placement of the erector spinae **(A)** and multifidus (**B**); Right: The electrode placement of the anterior deltoid (**C**).

Surface recording electrodes were placed on the body surfaces of both anterior deltoids, the erector spinae, and the multifidus muscles. The tested skin was prepared by shaving, rubbing with sandpaper, and wiping with 75% alcohol. The erector spinae electrodes were placed 3 cm from the spinal process of the third and fourth lumbar vertebrae, while the multifidus electrodes were placed 2 cm from a line between the fifth lumbar vertebra and the first sacral vertebra spinal process. The reference electrode was attached outside of the recorded range (17) (Figure 5).

The participants were instructed to relax and maintain upright standing with their feet parallel and hip-width apart. Participants were instructed to, after hearing the “start” command, quickly complete a full range of bilateral shoulder flexion, avoiding trunk rotation and shrugging. The sEMG of the bilateral anterior deltoids, erector spinae, and multifidus were simultaneously collected, and a total of three trials were performed (17) (Figure 4).

##### 2.4.1.3 sEMG data analysis

To detect muscle burst onset time, a 40-Hz Butterworth high-pass filter was used to eliminate motion and heart-beat artifacts (18). All filtered raw sEMG signals were full-wave rectified and smoothed using a moving average of 50 ms time constant (19).

The Noraxon sEMG software automatically detected the burst onset of the muscles from the pre-processed sEMG data, which was then verified manually through visual inspection. The sEMG signals of the lumbar muscle channel during 150–300 ms before the anterior deltoid activated were retrieved as baseline sEMG signals, and mean background EMG activity was calculated. The burst onset of each lumbar muscle was identified when the amplitude of linear enveloped EMG values deviated by 3 standard deviations above the mean background EMG activity and lasted for a minimum of 50 ms (20, 21).

The relative activation time of lumbar muscles was further calculated as per the following, which was used to judge if lumbar muscles activated in advance or delayed activating (22):

Relative activation time of lumbar muscle = activation time of lumbar muscle - activation time of anterior deltoid.

Lumbar muscles activate in advance when the difference is negative, while they delay activating when the difference is positive (22).

#### 2.4.2 LBP assessment

LBP was assessed by visual analog scale (VAS), which has been confirmed to be reliable in assessing LBP and proven to predict disability (23). A 10-cm vernier was used, with scores on one side, and “0” and “10” at the two ends, where “0” meant no pain, whereas “10” meant most severe and unbearable pain. The participants utilized the vernier to rate their pain intensity on a continuous scale, which can provide more detailed information than a simple categorical rating.

#### 2.4.3 Questionnaire on athletic training performance

In terms of a questionnaire, it was important to include questions that can provide insight into athletic training performance reported by elite trampoline athletes and the sports coach. By asking about the frequency of absence from training and inability to complete training tasks normally due to lumbar injuries, as well as progress in sports technology in the last month, we gathered valuable information about how lumbar injuries may be affecting the athlete and their training (Table 4).

**Table 4:** Correlation between differences of VAS score and relative activation time of lumbar muscles before and after intervention.

	*r*							
	Relative to left anterior deltoid				Relative to right anterior deltoid			
	Left erector spinae	Left multifidus	Right erector spinae	Right multifidus	Left erector spinae	Left multifidus	Right erector spinae	Right multifidus
VAS	0.598	0.411	0.561	0.505	0.293	0.044	0.287	0.349
*P*	>0.05	>0.05	>0.05	>0.05	>0.05	>0.05	>0.05	>0.05

VAS, Visual analog scale.

### 2.5 Statistical analysis

SPSS 26.0 (IBM Corporation, Armonk, NY) was used for the statistical analysis. Due to the limited sample size, a non-parametric test was recommended. The difference of the VAS score and the relative activation time of the four lumbar muscles before and after the intervention was tested for significance by the Wilcoxon rank sum test. The correlation between the difference in the VAS score and the relative activation time of bilateral lumbar muscles before and after intervention was tested by Spearson correlation analysis. *P* < 0.05 was considered statistically significant.

## 3 Results

### 3.1 Relative activation time of lumbar muscles in modified RART

The activation times of the right lumbar muscles relative to the left/right anterior deltoid significantly decreased after the intervention (*P* < 0.05); the activation time of the left lumbar muscles relative to the left/right anterior deltoid also significantly decreased after the intervention, more so than the right (*P* < 0.01) (Table 2).

**Table 2:** Activation time of lumbar muscles relative to left/right anterior deltoid in modified RART.

	Before treatment (s)	After treatment (s)	*Z*	*P*
Relative to left anterior deltoid				
Left erector spinae	0.11 (0.08, 0.14)	0.03 (−0.02, 0.05)**	−2.803	0.005
Left multifidus	0.11 (0.10, 0.16)	0.04 (−0.02, 0.08)**	−2.803	0.005
Right erector spinae	0.11 (0.08, 0.14)	0.04 (−0.01, 0.06)*	−2.293	0.022
Right multifidus	0.11 (0.08, 0.15)	0.04 (−0.01, 0.08)*	−2.293	0.022
Relative to right anterior deltoid				
Left erector spinae	0.07 (0.05, 0.16)	0.01 (−0.05, 0.06)**	−2.599	0.009
Left multifidus	0.09 (0.07, 0.16)	0.04 (−0.05, 0.07)**	−2.599	0.009
Right erector spinae	0.08 (0.03, 0.18)	0.02 (−0.01, 0.06)*	−2.191	0.028
Right multifidus	0.08 (0.04, 0.19)	0.03 (0.00, 0.07)*	−2.191	0.028

* *P* < 0.05.

** *P* < 0.01.

### 3.2 VAS

The VAS of LBP was significantly decreased after the intervention (*P* < 0.05) (Table 3).

**Table 3:** VAS scores.

	Before treatment	After treatment	*Z*	*P*
VAS	4.00 (3.00, 5.25)	3.00 (1.00, 4.25)	−2.059	0.040

VAS, visual analog scale.

### 3.3 Correlation between VAS and relative activation time of lumbar muscles

There was no significant correlation between the differences in VAS scores and the difference in the relative activation times of any of the lumbar muscles either relative to the left or right anterior deltoid before and after the intervention (*P* > 0.05) (Table 4).

### 3.4 Questionnaire on athletic training performance

After the intervention, one athlete reported a decrease, whereas the coach reported that four athletes had a decrease in the frequency of absence from training. In the improvement in progress in sports technology, two athletes reported improvement, whereas the coach reported that three athletes had improved. In the frequency of inability to complete training tasks normally due to lumbar injuries, the coach reported that three athletes had a decrease while one athlete had an increase (Table 5).

**Table 5:** Self-assessment/assessment of the elite trampoline athletes and the sport coach on athletic training performance.

Case	Age (years)	Gender (M/F)	Duration of training (years)	Duration of LBP (years)	Self-assessment of the elite trampoline athletes						Assessment of the trampoline sport coach					
					Frequency of absence from training in the last month		Progress in sport technology in the last month		Frequency of inability to complete training tasks normally due to LBP in the last month		Frequency of absence from training in the last month		Progress in sport technology in the last month		Frequency of inability to complete training tasks normally due to LBP in the last month	
					b	a	b	a	b	a	b	a	b	a	b	a
1	29	M	24	4	4	3	1	1	3	3	3	3	1	2	3	3
2	19	F	14	3	4	4	2	2	3	3	4	4	2	2	2	3
3	24	M	19	2	4	4	2	2	3	3	4	4	2	2	3	3
4	16	F	11	2	4	4	2	2	4	4	4	4	3	3	4	4
5	17	F	13	2	4	4	2	2	4	4	4	4	1	2	3	3
6	25	M	20	4	4	4	2	2	3	3	4	4	2	3	3	4
7	19	M	14	2	4	4	2	2	4	4	3	4	2	2	4	4
8	23	M	18	2	4	4	1	2	3	4	3	4	2	2	3	4
9	23	M	18	2	4	4	2	2	4	4	3	4	2	2	3	3
10	23	M	18	2	4	4	2	4	4	4	3	4	2	2	4	3

M, male; F, female; b, before; a, after; LBP, low back pain.

Frequency of absence from training in the last month: 1, always; 2, frequent; 3, occasional; 4, never.

Progress in sport technology in the last month: 1, no progress; 2, a little progress; 3, some progress; 4, great progress.

Frequency of inability to complete training tasks normally due to LBP in the last month: 1, always; 2, ≥2 times/week; 3, <2 times/week; 4, never.

## 4 Discussion

The activation pattern of the lumbar muscles in the modified rapid arm rise test was assessed by sEMG, firstly to clarify whether elite trampoline athletes with LMS manifest delayed activation of lumbar muscles under IPI. Secondly, following an integrative rehabilitation program, the therapeutic effects on improving neuromuscular control of the lumbar muscles, reducing pain, and improving athletic performance in elite trampoline athletes with LMS were assessed using modified RART VAS and an athletic performance questionnaire.

### 4.1 Delayed activation of lumbar muscles under IPI

The activation times of trampoline athletes' lumbar muscles relative to either deltoid at baseline were all positive, indicating delayed activation under IPI. This verified that trampoline athletes with LMS had poor feedforward control of their lumbar muscles, which is consistent with the findings in common patients with chronic LBP (5, 7).

Previous research has focused on biomechanical risk factors of the spine and lumbar muscles of trampoline athletes (2, 3), but few have studied the neuromuscular control of the lumbar muscles, which was the focus of this study. The lumbar muscles should be activated in advance before one actively raises the arm, which is a feedforward mechanism of the postural muscles based on psychological anticipation under IPI (15).

Feedforward control of the lumbar muscles is often studied under external or internal postural interference (15, 17). Unlike catching a ball sports as an external postural interference, trampoline sports involve IPI. RART has been commonly used to evaluate the activation pattern of the lumbar muscles under IPI, in which the sEMG of the lumbar muscles would be recorded when the right arm is raised rapidly in a full range of motion (5, 17). In consideration for the characteristics of trampoline sports, we modified RART for these athletes. Our results helped us to learn more about the neuromuscular control of the lumbar muscles in athletic LMS. It can be speculated that the brain's perception of movement may be blurred in this condition (2, 8), which would negatively affect the subsequent trampoline acrobatic skills and, thus, athletic performance. Furthermore, this delayed activation pattern of the lumbar muscles would conversely increase the risk of lumbar injuries (9), and even exacerbate LMS (2).

### 4.2 Effectiveness of integrative rehabilitation

Most importantly, our results demonstrated the effectiveness of integrative rehabilitation in relieving LBP and improving neuromuscular control of the lumbar muscles in elite trampoline athletes with LMS, which is consistent with previous research findings in general patients with chronic LBP (4, 5, 7–9). Physiotherapy, kinesiotherapy, and complementary therapies have been recommended for the treatment of chronic non-specific LBP (24). Chronic low back pain (CLBP) is a highly prevalent, burdensome, and costly health problem in Western industrialized countries. Currently, the development of unimodal conventional medical approaches has not resulted in consistently and markedly improved efficacy, leading many patients to seek complementary and integrative therapies (CIT) for CLBP. Surveys support that CLBP patients are among the highest users of CIT (25). In China, CIT has also been widely used clinically in CLBP patients and works better than using a single method (6). However, a systematic review reported that it is unclear which treatments are most effective for which athlete populations. While exercise approaches generally reduce pain and improve function in athletes with LBP, the effect on return to sport is unknown. There is still a lack of evidence on the effectiveness of commonly used interventions for LBP in athletes (10).

Therefore, we combined several intervention methods from Western medicine and TCM to apply this protocol to elite trampoline athletes with LBP. This pilot study preliminarily confirms the efficacy of integrative therapy in alleviating LBP and improving neuromuscular control in the lumbar muscles.

Concerning relieving pain, ESWT has been reported to have superiority in relieving chronic LBP (26). Tui-na, or Chinese massage, has been strongly recommended to relieve acute and sub-acute pain while acupuncture relieves LBP in all time courses including acute, sub-acute, and chronic phases (27). We considered that elite trampoline athletes not only had CLBP, but would also have acute or sub-acute LBP due to their daily intensive athletic training. Therefore, we believed that massage and acupuncture would play their roles in both transient acute LBP and cumulative CLBP in our protocol. In terms of improving physical function, Tui-na also contributes to relaxing tensive muscles and tendons (28). Additionally, kinesiotherapy has been strongly recommended for the management of NLBP to coordinate and strengthen the paraspinal muscles, and increase the stability of the spine (24). Thoracic spine function exercise and cat-arching poses help to coordinate the paraspinal muscles and increase spinal flexibility, while bird-dog and dead bug exercises assist in strengthening the paraspinal muscles (28).

We believe that our integrative therapy incorporates the benefits of multiple therapies in both pain relief and physical function improvement and has good therapeutic potential in elite trampoline athletes with LMS. LBP has long been a leading cause of days lost from work for common patients (29). For athletes, return to sport is a common outcome measure in LBP rehabilitation (10). For our participants, we placed more emphasis on whether their athletic performance would improve after the intervention. The information from the questionnaire also reflects a preliminary practical value of integrative rehabilitation in athletic LMS.

### 4.3 Correlation between LBP relief and improved delayed activation of lumbar muscles

Our study did not find significant correlation between LBP relief and improved delayed activation of lumbar muscles, which was not in line with previous findings (30, 31). A widely accepted theoretical model of pain adaptation in NLBP is that the motor control strategy adopted by the lumbar muscles in response to pain is to reduce movement speed and limit movement range (32). The delayed activation of the multifidus is even worse in patients with NLBP, contributing to a vicious circle of LBP and reduced stability of the lumbar spine (30, 31). However, our results were not consistent with these previous findings. We speculate that this might be due to two reasons. This correlation was previously demonstrated in common patients with LMS or NLBP (30, 31). It remains unknown if this correlation also exists in elite trampoline athletes with LMS; their core physical function and injury factors differ from those of the general population with LMS or NLBP, so this correlation may not manifest or be significant. Conversely, due to the small sample size of this pilot study, further clinical studies with a larger sample size are needed to demonstrate this issue.

### 4.4 Practical implications

Our study is the first to use sEMG to evaluate elite trampoline athletes with LMS, to analyze the neuromuscular control of their lumbar muscles under IPI. We innovatively modified the RART, which simulates the IPI of trampoline sport. Our findings provide initial support for the utilization of this test to evaluate the feedforward control of lumbar muscles during acrobatic skills in trampoline sports. In addition, the integrative rehabilitation program in this study showed benefits in terms of pain relief, improved neuromuscular control of the lumbar muscles, and improved athletic training performance. Our protocol could be further investigated in elite trampoline athletes or in athletes of other sports, especially those involving IPI.

### 4.5 Limitations and further recommendations

There are some limitations of this study that should be noted. First, the small sample size might limit the generalizability of the findings. Second, the lack of a control group made it difficult to rule out potential confounding factors. Finally, although the delayed activation of lumbar muscles was improved after intervention, it still existed in some participants. This encouraged us to further tailor the exercise type and dose, as trampoline athletes have unique movement patterns (21).

Future investigations or randomized-controlled trials with a larger sample size are needed to verify the efficacy of our integrative therapy in elite trampoline athletes with LMS, or its superior efficacy compared to a single therapy. Moreover, the questionnaire assessed by the athletes themselves and the coach needs to be modified and further developed and tested for reliability and validity, which could provide a more targeted scientific evaluation of the athletic training performance of elite trampoline athletes after treatment of LMS.

## 5 Conclusion

Elite trampoline athletes with LMS had delayed activation in their lumbar muscles under IPI. Integrative rehabilitation was effective for LBP relief and neuromuscular control of the athlete's lumbar muscles, and potentially impacted positively on their athletic training performance. Future studies with a larger sample size are needed to verify the efficacy of our integrative rehabilitation in elite trampoline athletes with LMS. Our protocol could be also investigated in athletes of other sports, especially those that involve IPI.

## Data Availability

The original contributions presented in the study are included in the article/**Supplementary Material**, further inquiries can be directed to the corresponding authors.
