# Supplementary material for: Integrative rehabilitation in the treatment of lumbosacral muscle strain in elite trampoline athletes: a pilot study
Source: Front Sports Act Living. 2024 Jul 9;6:1383228. doi: 10.3389/fspor.2024.1383228 (PMC11263099; doi:10.3389/fspor.2024.1383228)
Supplement: Supplementary file 1 [file Datasheet1.pdf]

Supplementary Material:

The indications and illustration of spine function training

Integrative rehabilitation in the treatment of lumbosacral muscle strain in elite trampoline athletes: a pilot study

Jia-Yin Ma M.S.[1†], Jia-Jia Wu M.D., PhD.[1,2†], Jing Zhang M.S.[7], Qing Zhao M.D.[1], Feng-Tao Shen, M.S.[1], Ling Feng M.D.[1], Guo-Hui Zhang M.D.[1], Yi Zhu M.D[3,4,5,6*], Jian-Guang Xu M.D., PhD.[1,2,3,5,6*]

*** Correspondence:** Jian-Guang Xu xjg@shutcm.edu.cn; Yi Zhu zhuyi_hsyy@hotmail.com

1 Thoracic spine exercise

1.1 Starting position

Supine on the bed with the foam roller under the lower segment of the thoracic spine. Bilateral knees were flexed 90°and the feet were flat on the bed. Bilateral hands were put behind of the head (Supplementary Figure 1).

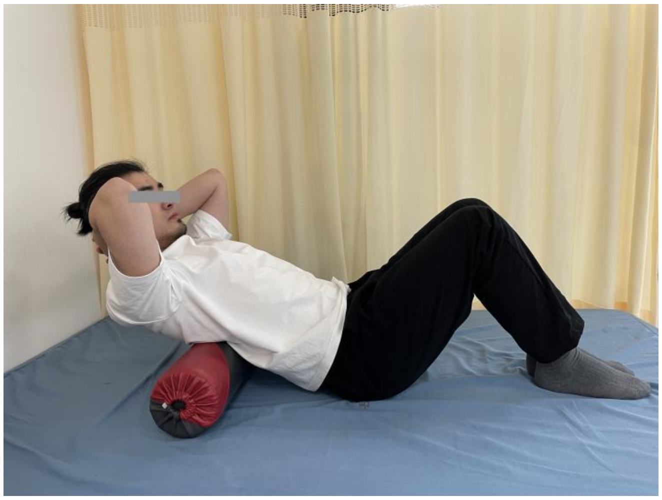

Supplementary Figure 1

1.2 Indications of the exercise

Aided with the foam roller rolling forward and backward, extend the thoracic spine when inhaling slowly, and flex the thoracic spine when exhaling slowly. Repeat this procedure which lasts for 5 minutes (Supplementary Figure 2).

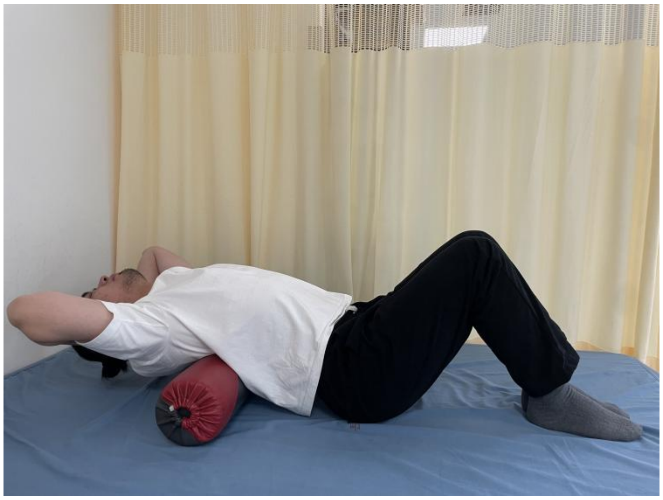

Supplementary Figure 2

2 Cat arching exercise

2.1 Starting position

Kneel on 4 points with bilateral knees and palms on the bed. The knees were kept hip-width apart while the palms were kept shoulder-width apart. Both the lumbar and abdominal muscles were kept contracted to keep the spine and pelvis in neutral position. Excessive flexion or extension of the spine should be avoided (Supplementary Figure 3).

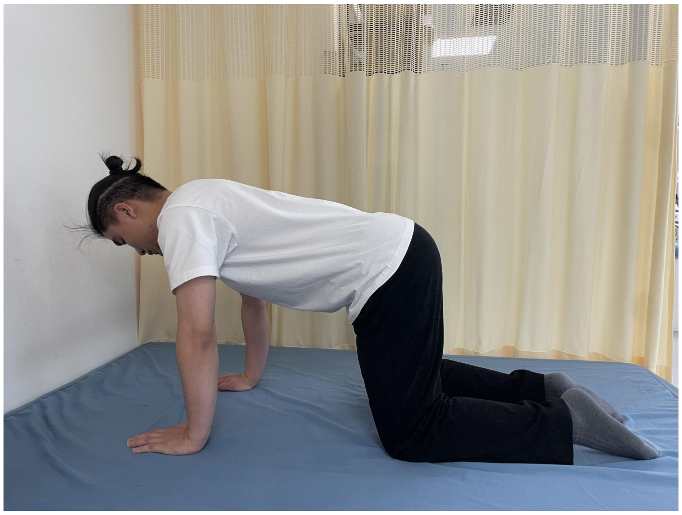

Supplementary Figure 3

2.2 Indications of the exercise

Extend the spine gradually with the eyes looking upward when inhaling slowly. Keep this position for 5s (Supplementary Figure 4(A)). And flex the spine gradually with two palms pressing the bed firmly, and the jaw and hip being lowered (Supplementary Figure 4(B)). The range of extension or

flexion the spine depened on each subject's endurance. Three groups should be done, of which involves 10 repetitions each group .

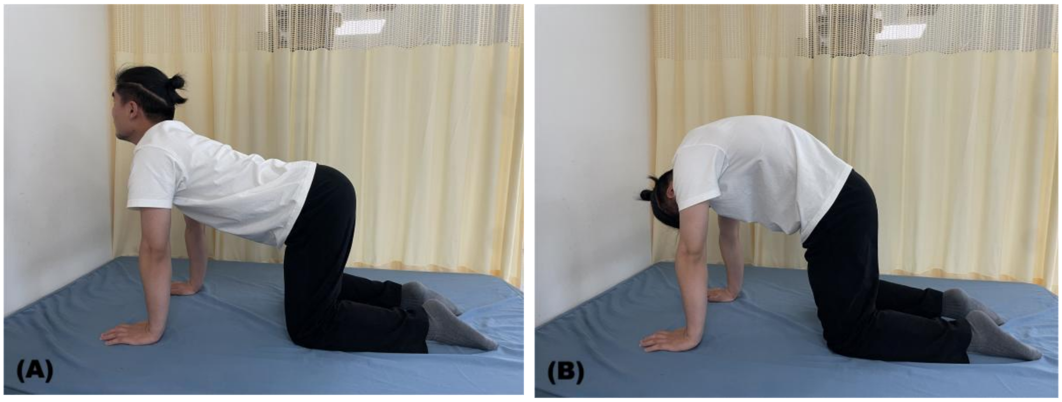

Supplementary Figure 4

3 Bird-dog exercise

3.1 Starting position:

Kneel on 4 points with bilateral knees and palms on the bed. The knees were kept hip-width apart while the palms were kept shoulder-width apart. Both the lumbar and abdominal muscles were kept contracted to keep the spine and pelvis in neutral position (Supplementary Figure 5). Excessive flexion or extension of the spine should be avoided.

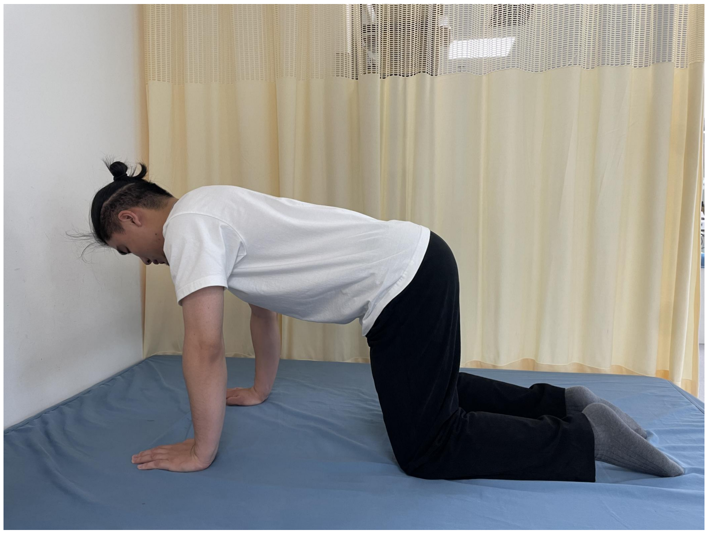

Supplementary Figure 5

3.2 Indications of the exercise

3.2.1 The hip of either side was slowly extended with the knee extended. Meanwhile, the shoulder of the opposite side was flexed with the elbow extended. The raised lower and upper limbs were both kept parallel with the bed. This posture was maintained for 5s, which could be prolonged as progression upon the physical therapist. The head and scapula should be kept in neutral and stable, and The hip rotation should be avoided (Supplementary Figure 6).

3.2.2 Return to the starting position (Supplementary Figure 5) and repeat the same movement in the other side.

Three groups should be done on each side, of which involves 10 repetitions each group.

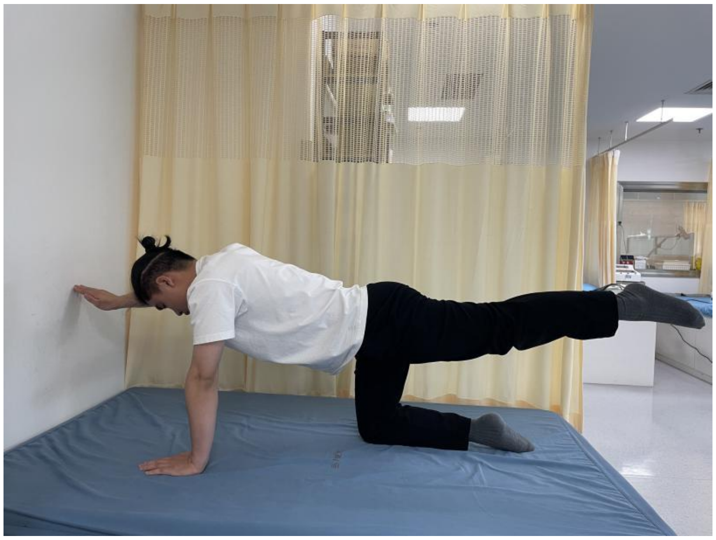

Supplementary Figure 6

4 Dead-bug exercise

4.1 Starting position

Supine on the bed with bilateral upper and lower limbs lifted. The shoulders should be 90°flexed with the elbows fully extended. Both the hips and knees should be 90°flexed. The bottom of the hip was slightly off the bed while the back was sticked to the bed (Supplementary Figure 7).

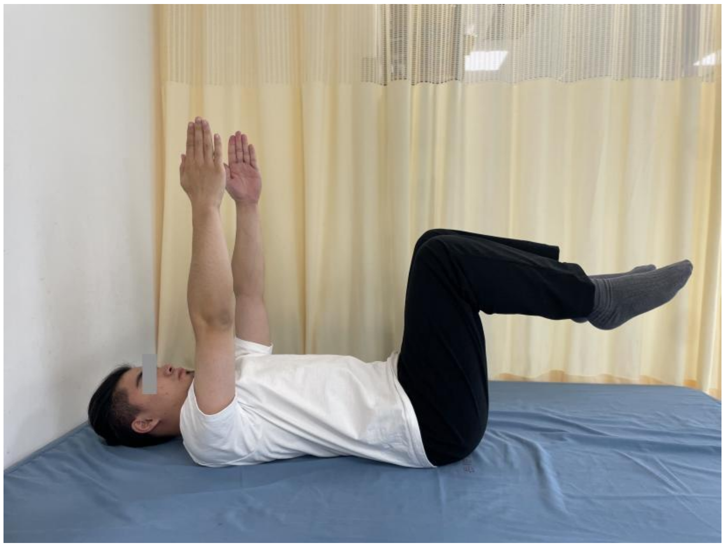

Supplementary Figure 7

4.2 Indications of the exercise

4.2.1 Flex the shoulder of the either side, and extend the hip of the opposite side, to keep the upper and lower limbs parallel to the bed. The movement should be slow, and the trunk should be kept stable. When maintaining in this posture, the lumbar and abdominal muscles were kept contracted and the back was kept sticked to the bed. This posture was maintained for 5s, which could be prolonged as progression upon the physical therapist (Supplementary Figure 8).

4.2.2 Return to the starting position (Supplementary Figure 7), and repeat the same movement in the other side.

Three groups should be done on each side, of which involves 10 repetitions each group.

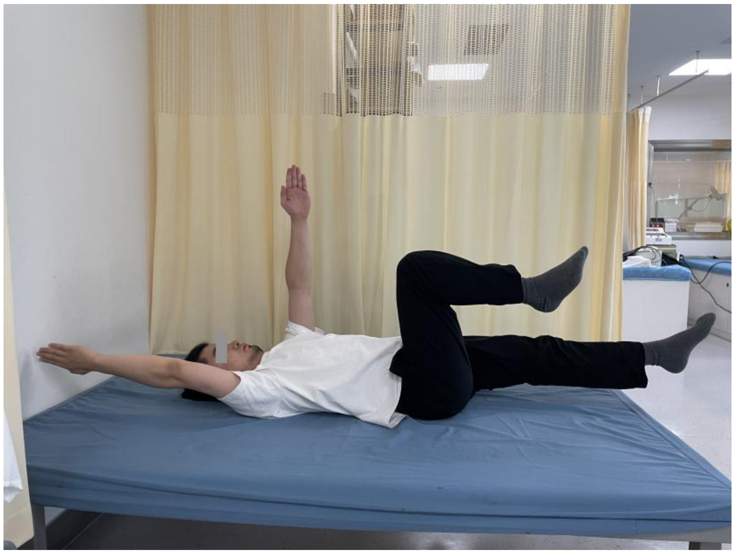

Supplementary Figure 8

Supplementary figure legends

Supplementary Figure 1 Starting position of thoracic spine exercise

Supplementary Figure 2 Illustration of thoracic spine exercise

Supplementary Figure 3 Starting position of cat arching exercise

Supplementary Figure 4 (A) illustration of cat arching exercise when flexing the spine (B) illustration of cat arching exercise when extending the spine

Supplementary Figure 5 Starting position of bird-dog exercise

Supplementary Figure 6 Illustration of bird-dog exercise

Supplementary Figure 7 Starting position of dead-bug exercise

Supplementary Figure 8 Illustration of dead-bug exercise
